# TCA Cycle-Mediated Generation of ROS Is a Key Mediator for HeR-MRSA Survival under β-Lactam Antibiotic Exposure

**DOI:** 10.1371/journal.pone.0099605

**Published:** 2014-06-16

**Authors:** Roberto R. Rosato, Regina Fernandez, Liliana I. Paz, Christopher R. Singh, Adriana E. Rosato

**Affiliations:** 1 Center for Molecular and Translational Human Infectious Diseases Research, Houston Methodist Research Institute, Houston, Texas, United States of America; 2 Department of Pathology and Genomic Medicine, Houston Methodist Hospital, Houston, Texas, United States of America; Rockefeller University, United States of America

## Abstract

Methicillin-resistant *Staphylococcus aureus* (MRSA) is a major multidrug resistant pathogen responsible for several difficult-to-treat infections in humans. Clinical Hetero-resistant (HeR) MRSA strains, mostly associated with persistent infections, are composed of mixed cell populations that contain organisms with low levels of resistance (hetero-resistant HeR) and those that display high levels of drug resistance (homo-resistant HoR). However, the full understanding of β-lactam-mediated HeR/HoR selection remains to be completed. In previous studies we demonstrated that acquisition of the HoR phenotype during exposure to β-lactam antibiotics depended on two key elements: (1) activation of the SOS response, a conserved regulatory network in bacteria that is induced in response to DNA damage, resulting in increased mutation rates, and (2) adaptive metabolic changes redirecting HeR-MRSA metabolism to the tricarboxylic acid (TCA) cycle in order to increase the energy supply for cell-wall synthesis. In the present work, we identified that both main mechanistic components are associated through TCA cycle-mediated reactive oxygen species (ROS) production, which temporally affects DNA integrity and triggers activation of the SOS response resulting in enhanced mutagenesis. The present work brings new insights into a role of ROS generation on the development of resistance to β-lactam antibiotics in a model of natural occurrence, emphasizing the cytoprotective role in HeR-MRSA survival mechanism.

## Introduction

*Staphylococcus aureus* is an important pathogen responsible for a number of diseases ranging from skin and soft tissue infections to life-threatening endocarditis, both in hospitals and community settings [1]–[3]. The primary target of β-lactam antibiotics are penicillin binding proteins (PBPs), which are involved in the last stages of peptidoglycan biosynthesis [4]. β-lactam resistance in MRSA involves the acquisition of PBP2a, a protein encoded by the *mecA* gene, that complements the four native staphylococcal PBPs (PBP1–4) once they have been inactivated [5], [6]. Expression studies showed that high level of *mecA* expression not always correlates with higher level β-lactams resistance [7]–[10]. The main characteristic of the majority of clinical MRSA isolates associated with both hospital and community associated infections is their heterogeneous expression to β-lactam antibiotics. Heteroresistant MRSA strains are composed of mixed cell populations containing both organisms in which the majority of the cells express resistance to low concentrations of oxacillin [heterotypic resistance (HeR)], with a minority of cells (≤ 0.1%) expressing resistance to a greater concentration and displaying high levels of drug resistance [i.e., ≥ 256 µg/ml; homotypic resistance (HoR)] [11]–[13]. The evidence that sub-minimal inhibitory concentrations (MICs) of antibiotics can play an important role in the generation and dissemination of antibiotic resistance has begun to emerge in recent years [14], [15]. We have demonstrated that exposure of heteroresistant (HeR)-MRSA clinical strains to sub-inhibitory concentrations of β-lactams results in the selection of the highly-homoresistant (HoR) phenotype through a mechanism involving both increased *mecA* expression and induction of the SOS response [12]. In these strains inhibition of PBP1 (cell wall component) was linked to SOS activation and enhanced mutagenesis in response to exposure to β-lactam antibiotics [16]. Moreover, in recent studies we demonstrated that β-lactam-mediated HeR/HoR selection is associated with increased expression of genes that generate acetate, suggesting that acetate generation is one of the main sources supplying the tricarboxylic acid (TCA) cycle activity [17]. In fact, viable inactivation of the TCA cycle abolished the capacity of SA13011-HeR to become highly resistant in the presence of β-lactam antibiotics [17], indicating that β-lactam-mediated HeR to HoR selection in MRSA strains is linked to specific metabolic changes including a significant increase in TCA cycle intermediates and a simultaneous decrease in fermentative pathways [17].

Reactive oxygen species (ROS) can damage DNA, RNA, proteins and lipids resulting in cell death when the level of ROS exceeds an organism detoxification process and repair [18], [19]. Despite these effects, bacteria growing under aerobic conditions generate endogenous ROS as a metabolic by-product [20]. It has been suggested that bactericidal antibiotic drugs enhance (ROS) formation by utilizing internal iron from iron-sulfur clusters to promote Fenton-mediated hydroxyl radical formation [19], [21]. In this mechanism described for Enterobacteriacea [19], [22] the primary interactions stimulate oxidation of NADH via the electron transport chain, emphasizing the TCA cycle-dependent up-regulation of respiration as a significant source of antibiotic-induced oxidative stress and antimicrobial lethality [19]. Other studies have questioned the concept of ROS-mediated cell death as a unified mechanism of killing [23], [24]. Moreover, studies in *E. coli* have described that gyrase inhibitors induce oxidative damage and death by a mechanism involving iron misregulation which drives the generation of highly destructive hydroxyl radicals via the Fenton reaction [25]. Paradoxically, generation of ROS may also have a protective role, as suggested by the observation that *E. coli* treated with plumbagin, a superoxide generator metabolite, showed increased resistance to bleomycin [26], supporting the notion that antimicrobial-mediated ROS may have opposing functions during lethal stress. In other words, generation of ROS may contribute to antibiotic lethality when the oxidative stress is lethal whereas in a context of sub-lethal stress, it can contribute to enhanced mutagenesis and survivability. In the latter, it has been shown that in *E.coli* sub-inhibitory concentrations of β-lactams can cause ROS production and PolIV-dependent mutagenesis; in this model, β-lactam antibiotics induce the RpoS stress regulon which is required to diminish the control of DNA fidelity by depleting MutS [27].

Based in these observations, we hypothesized that ROS formation plays a role in the β-lactam-mediated increased mutagenesis/SOS response associated to the HeR/HoR selection. The present study addresses this question highlighting a new cyto-protective mechanism played by β-lactam-mediated ROS generation in MRSA clinical strains.

## Materials and Methods

### Bacterial Strains, antibiotics and growth conditions used in this study

Clinical MRSA strain SA13011-HeR and derivatives are shown in Table 1. SA13011, a prototype of a heteroresistant MRSA strain previously described [12], [13], [28], is characterized as OXA susceptible and *mecA* positive [12], [28]. For this study, isogenic heteroresistant SA13011 strain (HeR; OXA MIC: 2 µg/ml) and its highly homoresistant methicillin-resistant derivative, SA13011-HoR; OXA MIC: 256 µg/ml) were used. These MRSA strains were identified as ST5, SCC*mec* type II, spaType 2, TJMBMDMGMK [28]. The mutants *lexA*, *acnA* (TCA-cycle) were constructed as previously described [12], [16], [29].

**Table 1:** Strains, plasmids, and primers used in this study.

Strain or plasmid	Description	Reference
SA13011-HeR	Heterogeneous-*mecA*(+) oxacillin susceptible	This study; [12]
SA13011-HoR	SA13011-HeR + OXA (0.5 µg/ml); SA13011 homogeneous derivative	This study; [12]
LMR-15	(SA13011Δ*acnA*::tetM)	[17]
LMR-16	(SA13011ΔacnA::tetM) OXA (0.5 µg/ml); LMR-15- homogeneous derivative	[17]
LMR-HeR	SA13011 HeR+pAD2 (pAD1-*lexA*-G94E)	[12]
LMR-HoR	SA13011 HeR+pAD2 +OXA (0.5 µg/ml)	[12]

Primers used in real-time RT-PCR assays		
*umuC*-F	TGCGAGTGTTTCTTGTATTG	
*umuC*-R	CCCTGTCTTGATGCCTAA	
*citB*-F	GGAAGAATGATGGATTTTGAATGTATG	
*citB*-R	GAAGTTTCAGATACCGTTGATAGTG	
*sodM*-F	GATGTTTGGGAGCATGCCTACTATC	
*sodM*-R	TTGCTGCTTGGTATAATTCATCAA	
*sodA*-F	GCCCAACCTGAACCAAAGCGTGC	
*sodA*-R	CTGGGAGTTACTTTCACCAAACTC	
*katA*-F	CTTTATGGATGTTGAACAAGCTGCG	
*katA*-R	CCTAATCGATATCTTTGCGCATCGCC	
*trxB*-F	TCCAGGCGGTCAAATGGCTAATACAGAAGA	
*trxB*-R	TTCGCCTTTATCTTCTACAGATTTAATATC	
16S-F	TCCGGAATTATTGGGCGTAA	
16S-R	CCACTTTCCTCTTCTGCACTCA	

All the antibiotics and chemicals used in this study including oxacillin (OXA, used at concentrations of 0.5 µg/ml), chloramphenicol (10 µg/ml), and tetracycline (5 µg/ml), were purchased from Sigma-Aldrich (St. Louis, MO); H_2_0_2_, thiourea, and 2-2′ bipyridyl were from Thermo-Fisher Scientific (Waltham, MA). The MIC of oxacillin was determined with Etest strips (BioMérieux, Crappone, France) according to the manufacturer's instructions.

Selection of SA13011 from the heterotypic (HeR) to the homotypic (HoR) resistance phenotype was performed as we previously described [12]. Briefly, bacteria were grown overnight in 5 ml LB broth without antibiotic, diluted to an OD_600_ of ∼0.025 in 300 ml LB broth, with or without 0.5 µg/ml OXA, and grown at 37°C with shaking (180 rpm). The OD was monitored every hour for up to 35 h. β-lactam-mediated HeR to HoR selection was verified by plating the cells onto an OXA gradient plate with a concentration ranging from 0 to 128 µg/ml. OXA MICs were determined by E-test (AB Biodisk, Solna, Sweden). To determine the functional role of β-lactam-induced ROS production, cells were incubated as described in the presence of ROS scavenger thiourea (120 mM) or the Fenton reaction inhibitor 2-2′bypyridil (0.25 mM).

### Analysis of Gene Expression by Real-Time RT-PCR

RNA extractions for real-time RT-PCR were performed as previously described [12], [30]. Total RNA was extracted using a RNeasy isolation Kit (Qiagen, Valencia, CA); all RNA samples were analyzed by A_260_/A_280_ spectrophotometry and gel electrophoresis to assess concentration and integrity, and cleaned of potential DNA contamination by treating them with DNAse per manufacturer recommendations (Ambion, Life Technologies, Austin, TX). Real-time RT-PCR analysis was done using the SensiMix SYBR One-Step kit (Quantace/Bioline, Taunton, MA) according to the manufacturer's protocol. Gene expression was compared according to the CT values converted to fold change with respect of a sample considered as reference (value  = 1) using log2–(ΔΔCt). The change (n-fold) in the transcript level was calculated using the following equations: ΔCT  =  CT(test DNA) − CT(reference cDNA), ΔΔCT  =  ΔCT(target gene) − ΔCT(16S rRNA), and ratio  = 2−ΔΔCT [31]. The quantity of cDNA for each experimental gene was normalized to the quantity of 16S cDNA in each sample as determined in a separate reaction. Each RNA sample was run in triplicate. Values represent the means of at least three biological replicates ± standard error of the mean (SEM), sampled in triplicate to minimize error by inter- and intra-samples. Differences between the mean values were analyzed using a one-way analysis of variance (ANOVA). A P value of <0.01 was considered statistically significant. Relative fold change values of specific mRNA in samples vs. corresponding reference value  = 1 are shown on the vertical axis. Oligonucleotide primers are shown in Table 1.

### Determination of mutation rate

Mutation frequencies for resistance to rifampicin were determined during OXA-mediated HeR/HoR selection ± thiourea (120 mM) and/or ± 2-2′bypyridil (0.25 mM). Inoculated flasks were incubated at 37°C with shaking at 145 rpm; aliquots of 100 µl were taken at different time intervals (e.g., 6, 27, and 33 hours) as previously described [13], [16]. All of the variants were selected on TSA plates containing rifampicin (200 µg/ml) and TSA plates using serial dilutions to determine CFU/ml. Mutation frequencies were expressed as the number of rifampicin-resistant mutants recovered as a fraction of the viable count. Three independent cultures were sampled in triplicate to minimize error caused by inter- and intra-sample variation.

### Detection of reactive oxygen species (ROS)

Levels of ROS were determined in cells collected after treatment, washed, resuspended in 1x PBS, and stained with the fluorescent reporter dye 3′-p-hydroxyphenyl fluorescein (HPF; InVitrogen-Life Technologies, Carlsbad). Fluorescence was measured by flow cytometry using a BD LSR II Flow Cytometer (BD, Franklin Lakes, NJ). Average fluorescence was normalized against a no-dye control. Data were analyzed using BD FACSDiva software. In addition, ROS production was also measured by using dihydrorhodamine 123 (DHR123) (Sigma-Aldrich) at a concentration 0.9 µg/ml during OXA-mediated HeR/HoR selection. DHR123 becomes fluorescent upon oxidation into rhodamine 123 in the presence of intracellular ROS [32].

### DNA Damage assay

DNA damage was determined in SA13011 MRSA cells grown under different conditions using a CometAssay (Trevingen, Gaithersburg, MD), as per manufacturer instructions, adapted following previous assays described in bacteria [33]. Briefly, after treatment with sub-inhibitory concentrations of β-lactams (i.e., OXA 0.5 µg/ml), cells were trapped in an inert agarose microgel placed on a microscope slide, deproteinized by incubation with lysing solution supplemented with lysostaphin 1 mg/ml and electrophoresed. DNA was stained with flurochrome SYBR Gold (excitation/emission is 496 nm/522 nm; Invitrogen) and visualized using a fluorescence microscope.

## Results

### Exposure to sub-inhibitory β-lactams induced early ROS production allowing HoR selection phenotype

**Figure 1:**
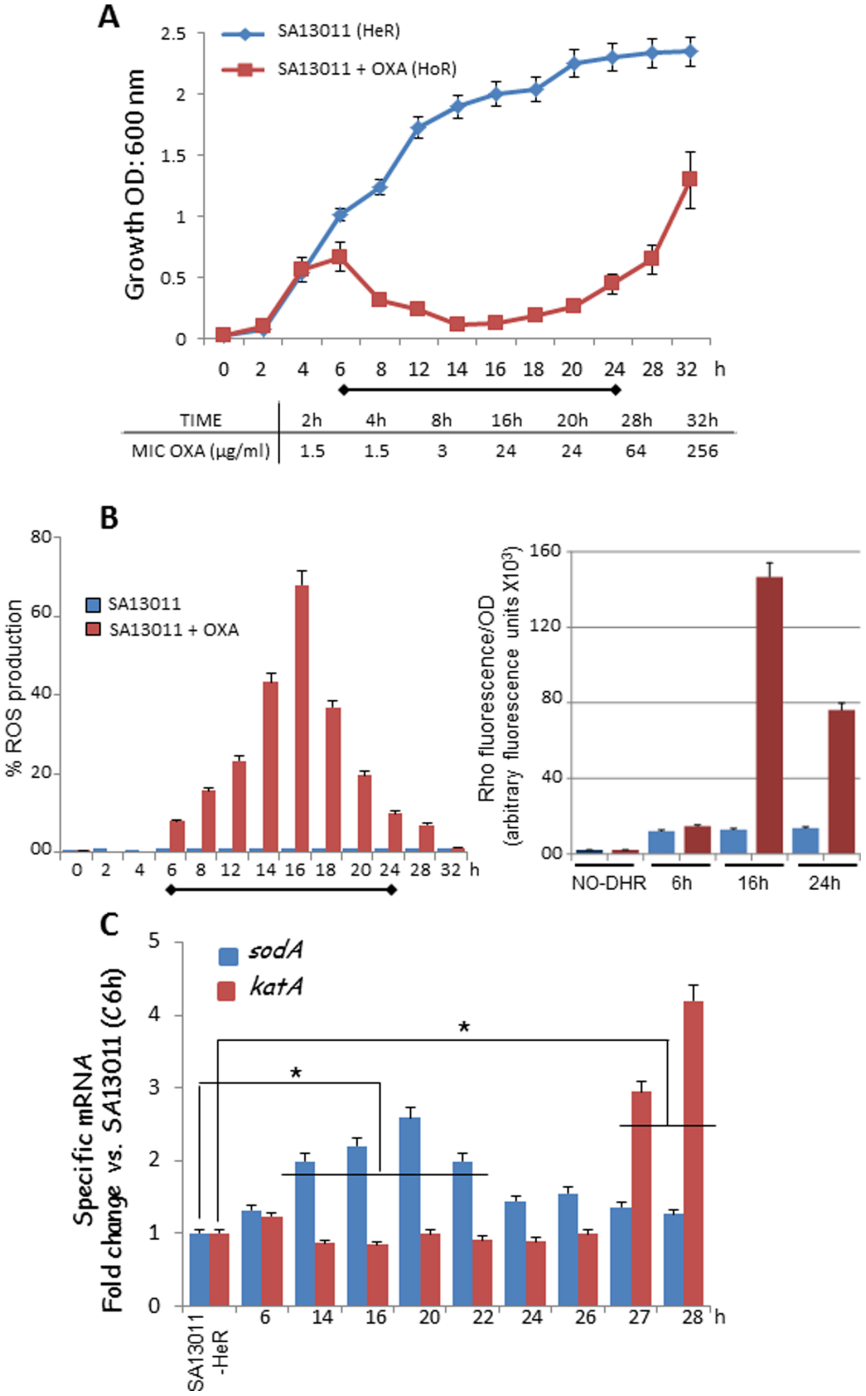
β-lactam-mediated ROS production and regulation. A) Time-course analysis of SA13011 HeR/HoR determined by optical density at 600 nm in SA13011-HeR growing + OXA (0.5 µg/ml); results represent the mean ± SEM of at least 3 experiments. Inset: values of MIC OXA (µg/ml) corresponding to the indicated time-points during the HeR/HoR selection. B) ROS measured in *SA13011-HeR* cells ± OXA (0.5 µg/ml); after collection at the indicated time-intervals, they were washed, resuspended in 1x PBS, stained with either the fluorescent reporter dye 3′-p-hydroxyphenyl fluorescein (HPF) or dihydrorhodamine 123 (DHR123), and analyzed by flow cytometry (HPF, left graph) or fluorescence at 500/550 nm wavelength excitation/emission (DHR 123, right graph), respectively. Results are representative of one experiment performed in triplicate; in the case of DHR 123, values represent arbitrary fluorescence units (x10^3^) per OD_600 nm_; additional experiments gave similar results. C) Gene expression levels of *sodA* and *katA* were determined by real-time RT-PCR using RNA from SA13011-HeR ± OXA (0.5 µg/ml) collected at the indicated time points. Relative fold-change values (SA13011-HeR reference value  = 1) of specific mRNAs are shown on the vertical axis. 16S rRNA was used as an internal control. *, *P*<0.01, statistically significant *vs.* corresponding reference value; horizontal lines cover samples significantly changed.

To investigate whether ROS production may be involved in the process of β-lactam-mediated HeR/HoR selection, sub-inhibitory concentrations of oxacillin (OXA; 0.5 µg/ml; MIC: 2 µg/ml) were added to cultures of SA13011 (*S. aureus* heteroresistant strain; clinical MRSA strain SA13011-HeR and derivatives are shown in Table 1), after which ROS formation was measured at different time points (Figure 1). The pattern of cell growth showed an initial increase in the optical density until 6 hours (OD_600 nm:_ 0.679, equivalent to 6.72×10^7^ CFU), followed by a lag phase of growth between 12 and 20 hours which corresponds to the killing of the susceptible cells of the HeR population. The subsequent increase in the OD [OD_600 nm:_ 0.19 (8.18×10^4^ CFU) - 1.3 (7.15×10^8^ CFU), 18 to 32 hours, respectively] represents the growth of the resistant HoR population (Figure 1A). Moreover, we observed that increase in OXA resistance paralleled the growth during the selection of the HoR population with MICs values to OXA ranging from 1.5 µg/ml (2 h) to 256 µg/ml (32 h; Fig 1A). The production of ROS was measured at different time points after addition of OXA (Figure 1B) using either the radical-sensitive dye hydroxyphenyl fluorescein (HPF; left panel) or dihydrorhodamine 123 (DHR123) While no changes in ROS levels were observed during the first 6 hours of growth, increasing amounts were detected between 8 and 16 hours (8–68%, respectively), followed by a marked declined returning to basal levels (18 to 32 h; Fig. 1B left panel). Consistently, similar results were observed with DHR123 at selected time points (Fig. 1B right panel). Production of ROS was not observed in cells grown in the absence of OXA at any time point (Fig. 1B).

In *S. aureus,* once a threshold level of ROS is sensed, a detoxification process starts that involves the activation of genes encoding superoxide dismutases (SOD) -A and -M (convert superoxides to oxygen and hydrogen peroxide), catalase A (converts hydrogen peroxide into water and oxygen), and thioredoxin reductase (catalyzes the reduction of thioredoxin) [34]–[36]. Expression analysis of the corresponding genes (*sodA*, *sodM, katA,* and *trxB*, respectively) showed no changes in *sodM* or *trxB* mRNA levels (data not shown), while both *sodA* and *katA* were significantly increased at earlier and later time intervals, respectively (Fig. 1C). The later increase in *katA* expression, notably when levels of ROS appeared to be under control, may reflect the fact that the first line of antioxidant activity is accomplished by superoxide dismutases; since the product of superoxide dismutation is hydrogen peroxide, levels of catalases may be only needed when it reaches a toxic level (i.e., later during the process of ROS regulation, 26-28 h; Fig. 1C) [35]. Together, and since changes in expression of antioxidant enzymes *sodA* and *katA* were both along the HeR/HoR selection process and temporally correlated with decreased amount of ROS detected in cells, these observations suggest a mechanistic involvement of these enzymes in the control and regulation of β-lactam-induced ROS production.

### β-lactam-induced ROS production is associated with DNA damage and activation of the SOS response in HeR-MRSA strains

**Figure 2:**
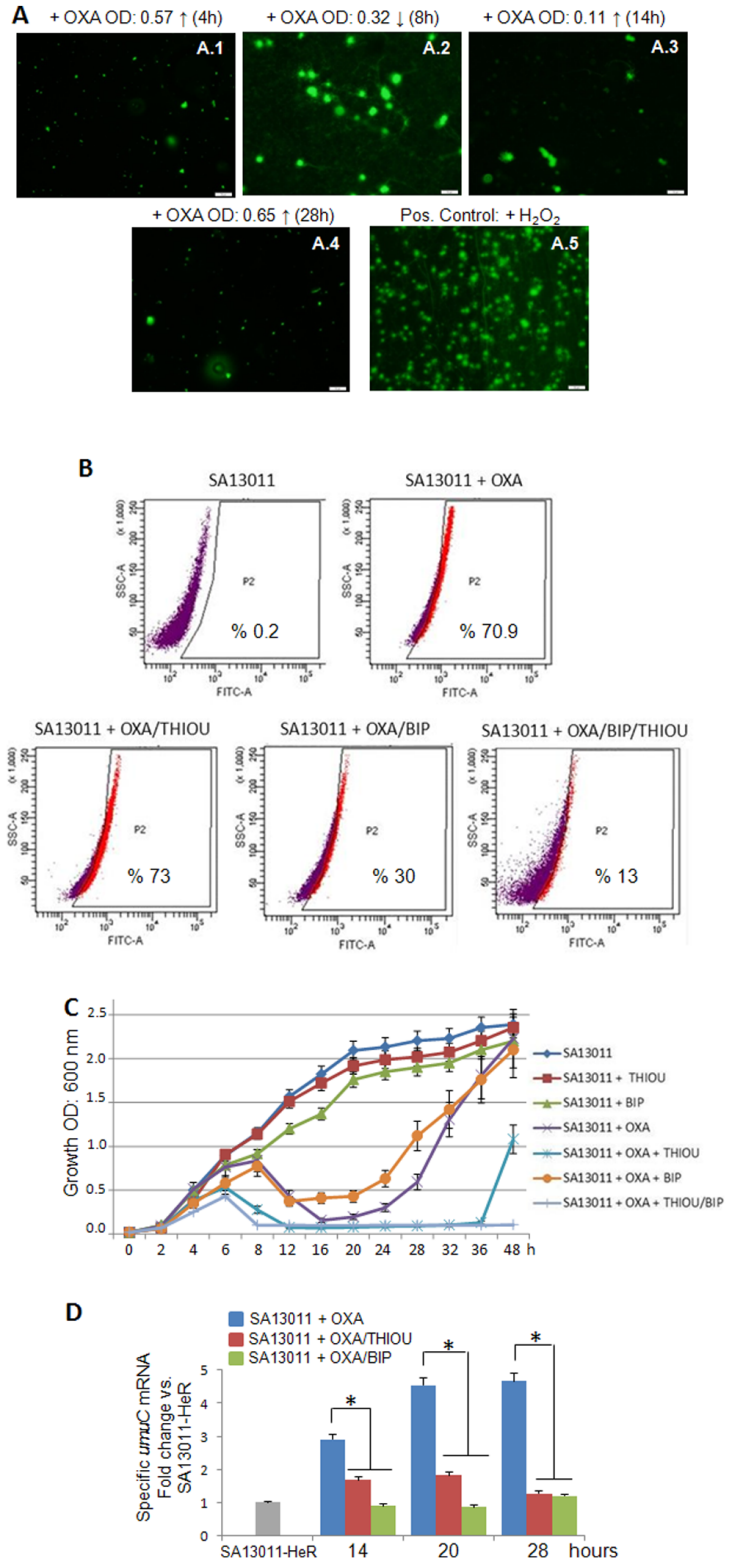
β-lactam-induced ROS production is associated with DNA damage and activation of the SOS response. A) DNA fragmentation was analyzed by CometAssay™. SA13011-HeR + OXA (0.5 µg/ml) was collected and processed as described in Methods; nucleoids showing either no signs (A1; OD600 nm: 0.57, 4 h) or fragmented DNA (spread, larger and more diffuse; A2-A3, OD600 nm: 0.32, 8 h; 0.11, 14 h; respectively) are shown. A.4: nucleoids corresponding to OD600 nm: 0.65, 28 h, without significant DNA fragmentation. A.5: SA13011-HeR cells were exposed overnight to 1 mM H2O2 and used as a positive control for DNA damage/fragmentation. B) ROS production following exposure to β-lactam ± thiourea (THIOU; radical scavenger) and/or 2,2′-bipyridyl (BIP; interferes with the Fenton reaction) was measured by flow cytometry using the reporter dye HPF in SA13011-HeR ± OXA (0.5 µg/ml) ± ThioU (120 mM), and ± BIP (0.25 mM), collected after 16 hours of exposure to these conditions; results are representative of one experiment performed in triplicate; additional experiments gave similar results. C) Time-course analysis of SA13011 HeR-HoR (OD600) in cells grown ± OXA (0.5 µg/ml), ± thiourea (120 mM, THIOU) or bipyridyl (0.25 mM, BIP). D) Quantitation of umuC mRNA expression levels (involved in activation of SOS response) by real-time reverse transcription (RT)-PCR using samples grown as indicated in C. Relative fold-change values (SA13011-HeR reference value  = 1) of specific mRNAs are shown on the vertical axis. 16S rRNA was used as an internal control. *, P<0.01, statistically significant vs. corresponding reference value.

DNA is substantially damaged when hydroxyl radicals are rapidly formed inside the cells [19]. To check for DNA integrity after exposure to β-lactams, DNA fragmentation was analyzed during SA13011 HeR/HoR selection (OXA 0.5 µg/ml) by Comet assay. While no signs of DNA damage were observed at early time-points (4 h, OD_600 nm_: 057, Fig. 2A1), nucleoids with fragmented DNA appeared concomitantly with maximal ROS formation [as shown in Fig. 1B, OD_600 nm_: 0.32 (8 h) and 0.11 (14 h), respectively; Fig. 2A2-3), which was followed by a progressive decrease in DNA damage at later time points (>24 h, OD_600 nm_: 0.65, 28 h; Fig. 2A.4). Cells were exposed to H_2_O_2_ (1 mM) as a positive control, displaying the characteristic fragmented DNA nucleoids (Fig. 2A.5). The SOS response is activated following genotoxic stress and initiates the highly-dynamic expression of SOS genes, including the error-prone DNA pol V (product of the *umuC* and *umuD* genes) that results in increased mutagenesis [22].

Taking into account i) recent observations in both *E. coli* and *S. aureus* showing the association of ROS-induced mutagenesis following treatment with sub-lethal concentrations of bactericidal antibiotics (e.g., ampicillin, norfloxacin) [12], [15]; and ii) our own studies demonstrating that β-lactam-mediated HeR/HoR selection in HeR-MRSA strains involves activation of the SOS response and increased mutation rate [12], we examined whether increased mutation rate may be associated with ROS production. To test this hypothesis, we used two antioxidant agents, thiourea, a potent radical scavenger [15], [37]–[39], and 2,2′-bipyridyl (BIP), a reagent that interferes with the Fenton reaction/ROS production [15], [18], [40]. Consistent with their respective functional roles, analysis of ROS formation (16 hours) in SA13011 showed that while OXA/thiourea-treated cells displayed no difference versus OXA-treated cells (i.e., 73% vs. 70.9%, respectively), decreased ROS were observed in the presence of BIP, either OXA/BIP or OXA/thiourea/BIP (30% and 13%, respectively; Fig. 2B). Neither thiourea nor BIP affected the growth rate of SA13011 in the absence of OXA; in contrast, in the presence of OXA, BIP displayed a rapid growth/selection (HoR), likely because by decreasing ROS production/mutagenesis, it may favor an advantageous fitness. In the other hand, thiourea produced a significant delay (∼12 hours), and the combination of all three agents (i.e., OXA/thiourea/BIP) impaired dramatically the HeR/HoR selection during the 48-hours time-interval (Fig. 2C). The phenotypic characteristics of these cells after selection were then evaluated by determining the mutation frequency in SA13011-HeR growing in the presence of OXA with/without thiourea or BIP (Table 2) at 6, 27, and 33 hours. We used the frequency of occurrence of rifampicin-resistant mutants as a marker for mutation frequency expressed as the ratio of rifampicin-resistant mutants recovered as a fraction of the viable count [12], [13], [16]. Exposure to sub-inhibitory concentrations of OXA (SA13011-HeR + OXA 0.5 µg/ml) determined a ∼5-log increase in mutation rates *vs.* the strain growing in the absence of OXA (SA13011-HeR) at the 33 h interval (i.e. 1.03×10^−3^ *vs.* 1.3×10^−8^, respectively; Table 2). By contrast, cells growing + OXA in the presence of anti-oxidants displayed significant decreases in the mutation rate, i.e. 6.0×10^−5^ (OXA/thiourea), effect even more pronounced in the presence of BIP (i.e., 4.6×10^−6^ and 1.1×10^−6^, OXA/BIP and OXA/thiourea/BIP, respectively; Table 2). Importantly, the reduction in mutation rate coincided with a reduction in the OXA-resistant phenotype: MIC >256 µg/ml (SA13011 + OXA) *vs.* 32 µg/ml (OXA/thiourea), 16 µg/ml (OXA/BIP), or 1.5 µg/ml (OXA/thiourea/BIP), the latter completely abolishing SA13011 capacity to achieve high levels of resistance under β-lactam exposure (Table 2). In line with this evidence, expression of *umuC* (SOS response), which increased during OXA-induced HeR/HoR selection, remained unchanged in the presence of either thiourea or BIP (Fig. 2D), further emphasizing the role of β-lactam-mediated ROS generation in the activation of the SOS response and increased mutagenesis. Together, these results support the concept that OXA-induced ROS production is a key mediator of β-lactam-mediated increased mutation rates and resistance.

**Table 2:** MICs and mutation rate analysis of SA13011 and LMR1 derivative.

Strains	MICs OXA (µg/ml)	Mutation rate	
SA13011-HeR	1.5	1.30×10^−8^	
SA13011-HeR + OXA	>256	1.03×10^−3^	
SA13011-HeR + ThioU	1.5	2.00×10^−8^	
SA13011-HeR + OXA/ThioU	32	6.00×10^−5^	
SA13011-HeR + BIP	1.5	2.76×10^−8^	
SA13011-HeR + OXA/BIP	16	4.60×10^−6^	
SA13011-HeR + OXA/ThioU/BIP	1.5	1.10×10^−6^	
LMR1-HeR	0.38	1.50×10^−8^	
LMR1-HoR	8	4.80×10^−7^	

Mutation frequencies are expressed as the number of antibiotic-resistant mutants recovered as a fraction of the viable count. Three independent cultures were sampled in triplicate to minimize error caused by inter- and intra-sample variation. OXA: oxacillin 0.5 µg/ml; ThioU: thiourea 120 mM; BIP: bipyridyl 0.25 mM.

### β-lactam-redirected TCA cycle activation is involved in ROS production

**Figure 3:**
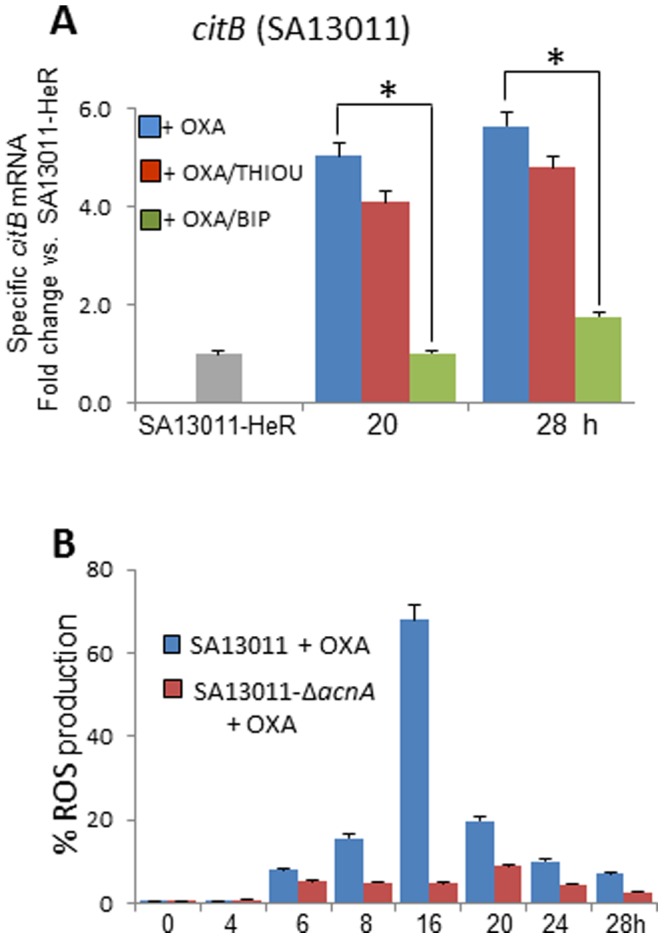
β-lactam-redirected TCA cycle activation is associated with ROS production. A) RNA from SA13011 + OXA (0.5 µg/ml) ± thiourea (THIOU, 120 mM), or ± bipyridyl (BIP, 0.25 mM) were used to quantitate TCA cycle-associated *citB* gene expression levels by real-time RT-PCR. Relative fold change values (SA13011-HeR_6h_ reference value  = 1) of specific mRNAs are shown on the vertical axis. 16S rRNA was used as an internal control. *, *P*<0.01. (B) ROS levels were determined in both *SA13011 (control) and* SA13011-Δ*acnA* mutant + OXA (0.5 µg/ml); cells were stained with the fluorescent reporter dye HPF and analyzed by flow cytometry to determine the percentage of cells displaying an increase in ROS production.

During exposure to β-lactams, HeR-MRSA redirects its metabolism to optimize TCA cycle energy production [17]. Expression analysis of the TCA cycle gene *citB/acnA*, which encodes aconitase, the second and main catabolic enzyme of the TCA cycle, clearly showed a marked induction during OXA-mediated HeR/HoR selection (Fig. 3A). Consistent with their function as either an ROS scavenger (thiourea) or inhibitor of the Fenton reaction/TCA cycle (BIP), the former displayed minimal effects on *citB* gene expression while addition of BIP inhibited its induction (Fig. 3A). To test whether HeR/HoR selection-associated ROS production may be dependent on the activated TCA cycle, ROS production was measured in SA13011-HeR Δ*acnA* mutant strain grown ± OXA (0.5 µg/ml) and compared against SA13011-HeR + OXA (0.5 µg/ml). As previously shown [17], no HeR/HoR selection was observed in SA13011-HeR Δ*acnA* growing in the presence of OXA (SA13011-HeR Δ*acnA* + OXA: MIC OXA: 1.0 µg/ml *vs.* >256, SA13011 + OXA). Importantly, impairment of the TCA cycle resulted in a significant reduction in ROS production at all tested time points (Fig. 3B). Furthermore, OXA-mediated selection from HeR to HoR did not take place under anaerobic conditions (data not shown), further emphasizing the pivotal role of an activated TCA cycle. Together these observations strongly support the notion that increased ROS levels are directly linked to a β-lactam-induced hyperactive TCA cycle.

### β-lactam-mediated ROS generation is associated to the triggering of the SOS response

**Figure 4:**
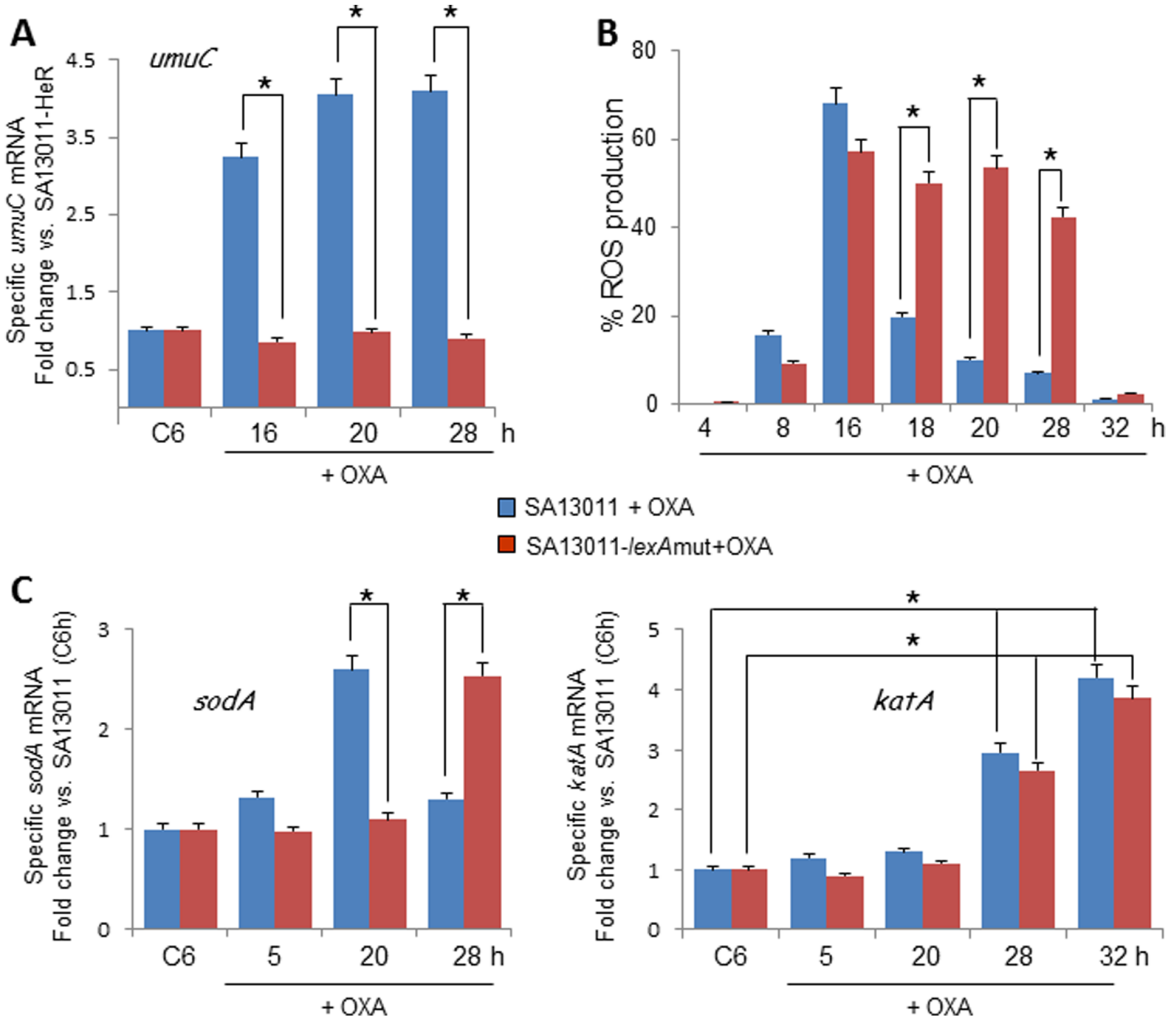
β-lactam-induced ROS production is associated to the triggering of the SOS response. Quantitation of *umuC* (A), *sodA* and *katA* (C) mRNA expression levels by real-time RT-PCR. RNAs were prepared from SA13011 and SA13011-lexAmut strains ± OXA (0.5 µg/ml) at the indicated time-intervals. Relative fold change values (SA13011-HeR_6h__growth_ reference value  = 1) of specific mRNAs are shown on the vertical axis. 16S rRNA was used as an internal control. *, *P*<0.01. Relative fold change values of specific SA13011 and SA13011-lexAmut strains + OXA *umuC* mRNA *vs.* the corresponding strains growing in the absence of OXA (reference value  = 1) are shown on the vertical axis. B) Levels of ROS were determined in *SA13011 (control) and* SA13011-lexAmut strains growing + OXA (0.5 µg/ml); cells were stained with the fluorescent reporter dye HPF and analyzed by flow cytometry to determine the percentage of cells displaying an increase in ROS production.

Previously, we have shown that β-lactam-induced *lexA/recA*-mediated SOS response is responsible for the increased mutation rate and selection of the highly-resistant HoR derivative in clinical HeR-MRSA strains [12]. To investigate the potential mechanistic association between ROS production and the SOS response, we used a SA13011-based *lexA* mutant [12]. In this SA13011 derivative strain the endogenous *lexA* chromosomal copy has been replaced with a modified form of the gene (G94→E) that results in a non-cleavable LexA and therefore uninducible SOS response (SA13011-*lexA*mut, strain LMR1; Table 1) [12]. When grown in the presence of β-lactams, LMR1 displayed significantly decreased growth [12], mutation rate (1.3 × 10^−8^/1.03 × 10^−3^ *vs.* 1.5 × 10^−8^/4.8 × 10^−7^, time-point 28 h, SA13011-HeR/HoR, LMR1-HeR/HoR, respectively), and MIC to OXA (>256 µg/ml *vs*. 8 µg/ml, SA13011-HoR/LMR1-HoR, respectively; Table 2). Expression analysis of *umuC* as an indicator of activation of the SOS response showed lack of induction in the SOS response-impaired LMR1 mutant strain exposed to OXA (Fig. 4A). Interestingly, ROS levels determined in both SA13011 and LMR1 strains grown in the presence of OXA followed a similar pattern up to 16 hours, after which ROS contents started to return to their basal level in the parental SA13011strain while remaining significantly elevated in LMR1 up to 28 hours (Fig. 4B). mRNA expression of *sodA* and *katA* were consistent with this pattern (Fig. 4C), i.e., *sodA* levels appeared induced in the LMR1 mutant several hours later (28 hours versus 20 hours in parental SA13011; Fig. 4C), which may explain the delay in ROS detoxification to baseline.

## Discussion

Heterogeneous MRSA strains can coexist as two populations of cells (HeR/HoR). These strains are frequently isolated from hospital and community and are mostly associated to either persistent or difficult-to-eradicate infections [12], [41]–[43]. In an effort to unveil the molecular mechanisms associated to heteroresistance in MRSA, a number of chromosomal factors have been characterized whose activities affect the level of resistance [44], [45]. Many of these genes are involved in cell wall biosynthesis and their study has given valuable insight into this pathway [45]. More recent lines of evidence regarding factors associated to Hetero-/Homo-resistance transition include a mutation in the diadenylate cyclase gene (*dacA*), a cyclase recently shown to synthesize the second messenger cyclic diadenosine monosphosphate (c-di-AMP), which influences methicillin resistance [46]. By analyzing an isogenic pair of MRSA strains that differed in both fitness and methicillin resistance, Dengler et al. found that a single mutation in the of *dacA*-gene correlated with decreased c-di-AMP levels, faster growth rate in the less resistant strain [47]. 2- In another interesting study the *mecA* determinant was introduced into a Methicillin Susceptible *S. aureus* (MSSA) either by a plasmid containing *mecA* or in the form of chromosomal SCC*mec*; in either case, the modified strain expressed heterogeneous resistance in which the highly resistant sub-populations, in addition to displaying increased levels of PBP2a, showed induction of the stringent stress response [48]. Importantly, several heterogeneously resistant MRSA clones that were tested could be converted to express the homoresistant phenotype by growing them in the presence of the stringent stress response inducer mupirocin [48].

We and others [11], [12], [44] have shown that the frequency at which highly resistant subclones (HoR) arise is a reproducible phenomenon and usually is above the rate of spontaneous mutation [11], [12], [44]. In this context, recent findings have demonstrated that certain mutations found on the *rpoB* gene (encodes the RNA polymerase β-subunit) are associated to heterogeneous-to-homogenous selection [49]. These observations suggest that a mechanism of mutagenesis is clearly associated to heteroresistance in MRSA; however, the factors leading to this mutational event have not been fully identified. In previous studies we determined that increased mutation rate during β-lactam-mediated HeR/HoR selection involved the induction of LexA/RecA-mediated SOS response [12]. Moreover, we identified unique metabolic features that were associated to HeR-MRSA undergoing β-lactam selection, most notably significant increase of TCA cycle intermediates and concomitant decrease of fermentative pathways [17]. Adaptative metabolic changes in HeR-MRSA undergoing β-lactam selection indicate that energy production is being redirected to supply the cell wall synthesis and metabolism [17]. In the present study, we show the mechanistic role played by generation of ROS in the acquisition of the highly resistant HoR phenotype, in a context in which increase in ROS levels during exposure to β-lactam appears as highly relevant.

Numerous observations in *E. coli* have linked ROS production with bactericidal action of antibiotics, pointing to antibiotics-induced TCA cycle- and respiratory chain-dependent ROS production as playing a role in cell death [19], [50]. Similarly, in *S. aureus*, co-incubation with BIP/thiourea has been shown to reduce the antimicrobial efficacy of daptomycin, moxifloxacin and OXA [51]. On the other hand, as mentioned before, in a context of sub-lethal oxidative stress, a protective role of ROS against cell death was also described in *E. coli* where treatment with the metabolic superoxide generators plumbagin or paraquat reduced the killing by oxolinic acid, kanamycin and ampicillin [26].

In our study, several important observations were made: 1- β-lactam-induced HeR/HoR selection is associated to oxidative stress mediated by production of ROS and DNA damage; 2- alleviation of β-lactam-mediated ROS production by 2-2′bypyridil suggest that the Fenton reaction is the major source of hydroxyl radicals; 3- the presence of both 2-2′bypyridil and thiourea drastically affects the capacity of generating mutations and, more importantly, the ability of HeR cells to acquire the highly homoresistant phenotype; 4- ROS production during β-lactam-mediated selection appears to be regulated by enzymes (namely dismutases and catalases) that protect HeR-MRSA from cell death and promote survival; 5- inactivation of TCA cycle regulation impaired ROS production, indicating the active contribution of metabolic adaptations to the HoR phenotype; and 6- the mechanism of β-lactam-stimulated mutagenesis in HeR-MRSA represents an effect that results from the conjunction between ROS production and SOS-induced response.

Several recent observations are in line with the present study. For example, works by Baharoglu et al. in *V.cholerae* demonstrated that exposure to sub-lethal concentrations of aminoglycosides (e.g. tobramycin) leads to ROS formation and DNA damage-mediated SOS response. In this work, the authors also established that, while the general stress regulator RpoS prevented oxidative damage in *E.coli*, it was rapidly degraded in *V.cholerae*, suggesting a differential degree of protection between species [52]. Furthermore, in agreement with our results pointing to a TCA cycle-dependent mechanism of ROS production, in *Staphylococcus epidermidis* was shown that exposure of a wild-type strain to sub-MIC oxacillin resulted in a significant increase in the production of ROS; moreover, no such increase was observed in a mutant with dysfunctional TCA cycle (ΔTCA) upon antibiotic challenge, suggesting oxacillin-mediated ROS production was TCA cycle-dependent [53]. In fact, the present results involving 2-2′bypyridil are consistent with recent evidence showing that, as an iron chelating agent, it inhibits not only the Fenton reaction and ROS production, but also the TCA cycle, as iron constitutes a critical co-factor of the TCA cycle enzyme aconitase [54]. Thus, reduction of the TCA cycle activity by either the aconitase null mutant or 2-2′bypyridil, provided strong support to the concept that metabolic changes associated with β-lactam mediated ROS production are critical components for the acquisition of the HoR phenotype in MRSA strains.

The present results integrate ROS production into our model where β-lactam-mediated re-direction of metabolism (activated TCA cycle) results in ROS generation and temporal DNA damage, both factors responsible of triggering the SOS response and enhanced mutagenesis, which in turn promotes survival in the presence of β-lactam antibiotics. Altogether, these observations demonstrate β-lactam-induced ROS production as a key mediator in the acquisition of the highly-resistant phenotype in clinical HeR-MRSA strains; importantly, they reveal new insights on the mechanisms of heteroresistance in clinical MRSA and expand target alternatives that may result in anti-infective therapeutic options.
